# Reciprocal Change of Cervical Spine after Posterior Spinal Fusion for Lenke Type 1 and 2 Adolescent Idiopathic Scoliosis

**DOI:** 10.3390/jcm12175599

**Published:** 2023-08-28

**Authors:** Kanji Mori, Jun Takahashi, Hiroki Oba, Tetsuhiko Mimura, Shinji Imai

**Affiliations:** 1 Department of Orthopaedic Surgery, Shiga University of Medical Science, Tsukinowa-cho, Seta, Otsu 520-2192, Shiga, Japan; simai@belle.shiga-med.ac.jp; 2 Department of Orthopaedic Surgery, Shinshu University School of Medicine, Asahi 3-1-1, Matsumoto 390-8621, Nagano, Japan; jtaka@shinshu-u.ac.jp (J.T.); oba1@hotmail.co.jp (H.O.); tettim3@yahoo.co.jp (T.M.)

**Keywords:** adolescent idiopathic scoliosis, cervical sagittal alignment, reciprocal change, SRS-22r, quality of life, cervical kyphosis, cervical hyperkyphosis, T1 slope, Lenke type 1, Lenke type 2

## Abstract

Reciprocal sagittal alignment changes after adolescent idiopathic scoliosis (AIS) posterior corrective surgery have been reported in the cervical spine, but the evidence is not yet sufficient. Furthermore, much remains unknown about the effects of cervical kyphosis on clinical outcomes in AIS. Forty-five consecutive patients (4 males and 41 females) with AIS and Lenke type 1 or 2 curves underwent a posterior spinal fusion, and a minimum of 24-month follow-up was collected from our prospective database. We investigated radiographic parameters and SRS-22r. Before surgery, cervical kyphosis (cervical lordosis < 0°) was present in 89% and cervical hyperkyphosis (cervical lordosis < −10°) in 60%. There were no significant differences in age, sex, or Lenke type between the hyperkyphosis and the non-hyperkyphosis groups. Although cervical lordosis increased significantly after surgery, cervical kyphosis was observed in 73% of patients 2 years after surgery. We found a significant correlation between Δthoracic kyphosis (TK) and Δcervical lordosis. Preoperative cervical kyphosis, ΔT1 slope, and ΔTK were independently associated factors for postoperative cervical hyperkyphosis. The cervical hyperkyphosis group had significantly lower SRS-22r domains. In AIS corrective surgery, restoring TK leading to a gain of T1 slope may lead to an improvement of cervical sagittal alignment. Remaining cervical hyperkyphosis after AIS surgery may affect clinical outcomes.

## 1. Introduction

Adolescent idiopathic scoliosis (AIS) is a relatively common three-dimensional spinal deformity [1]. AIS occurs in approximately 2–3% of the general population [2]. Patients with thoracic AIS typically have hypokyphotic thoracic spines compared to the same-aged normal population. Several studies have analyzed the relationship between lumbar lordosis (LL) and thoracic kyphosis (TK) [3,4,5,6]. Thus, the literature has described that restoring adequate TK is important to avoid a consequent loss of LL that could lead to flatback syndrome [7,8]. Although there are limited reports on cervical alignment in AIS compared to thoracolumbar alignment, it has attracted the attention of researchers in recent years. The incidence of cervical kyphosis in the AIS population is higher than in healthy individuals with a non-lordotic (straight, sigmoid, or kyphotic) cervical spine [9,10,11]. Reciprocal sagittal alignment changes after AIS posterior corrective surgery have been reported not only in the lumbar spine but also in the cervical spine [12]. Hayashi et al. [12] investigated the relationship between the perioperative cervical lordotic angle and other radiographic factors or clinical results in 51 AIS and identified independent risk factors for postoperative cervical hyperkyphosis. They found that the cervical lordotic angle increased significantly from preoperatively to 2 years after surgery. In addition, when postoperative cervical hyperkyphosis was defined as a cervical lordotic angle smaller than −10° at 2 years postoperative follow-up, preoperative small cervical lordotic angle and TK measurements were independent risk factors for postoperative cervical hyperkyphosis. However, the evidence of reciprocal sagittal alignment changes after AIS posterior corrective surgery is not yet sufficient. Furthermore, much remains unknown about the effects of cervical kyphosis on clinical outcomes such as quality of life (QOL) in AIS. However, considering the results of recent studies on adult spinal deformity, spinal alignment should have a significant impact on QOL in AIS as well.

The purpose of this study was to investigate the changes in cervical sagittal alignment (CSA) following surgical correction and the association between CSA, especially cervical hyperkyphosis, and QOL in postoperative patients with AIS using our prospectively collected data. Furthermore, we investigated the factors associated with postoperative cervical hyperkyphosis.

## 2. Materials and Methods

Consecutive patients with AIS, Lenke type 1 or 2 curves, who underwent surgical treatment between November 2017 and March 2020 and had a minimum of 24-month follow-up were collected from our prospective database. Patients underwent a posterior spinal fusion (PSF) without additional anterior release or prior spinal surgery. Exclusion criteria included (1) a follow-up period of less than 24 months, and (2) incomplete radiographical records, such as radiographs without the entire cervical spine.

**Figure 1:**
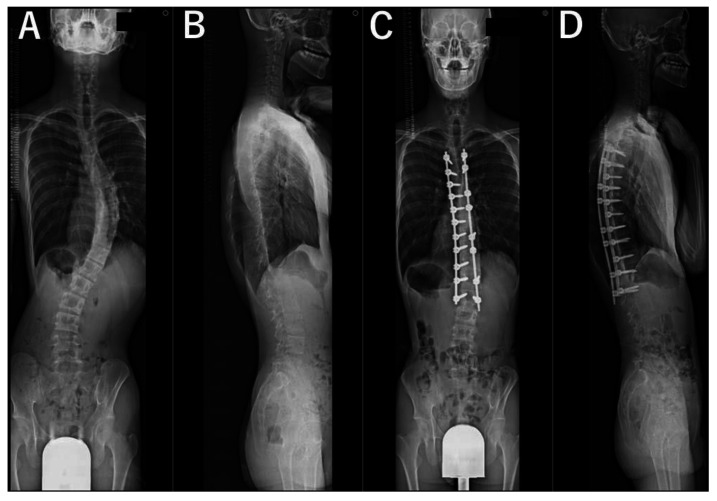
Pre- (**A**,**B**) and post- (**C**,**D**) operative radiographs of representative case with 16-year-old male patient. Cases of preoperative cervical kyphosis changed to postoperative cervical lordosis. Cervical lordosis (−5°, 18°), thoracic kyphosis (12°, 15°), lumbar lordosis (48°, 42°), T1 slope (11°, 22°), major thoracic curve (53°, 18°). (Preoperative, postoperative).

**Figure 2:**
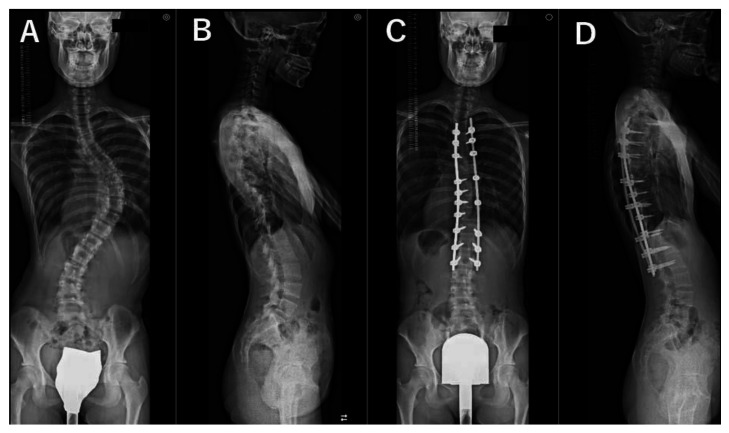
Pre- (**A**,**B**) and post- (**C**,**D**) operative radiographs of representative case with 17-year-old female patient. Cases of preoperative cervical hyperkyphosis changed to postoperative cervical kyphosis. Cervical lordosis (−19°, −8°), thoracic kyphosis (20°, 26°), lumbar lordosis (59°, 64°), T1 slope (11°, 12°), major thoracic curve (78°, 18°). (Preoperative, postoperative).

**Figure 3:**
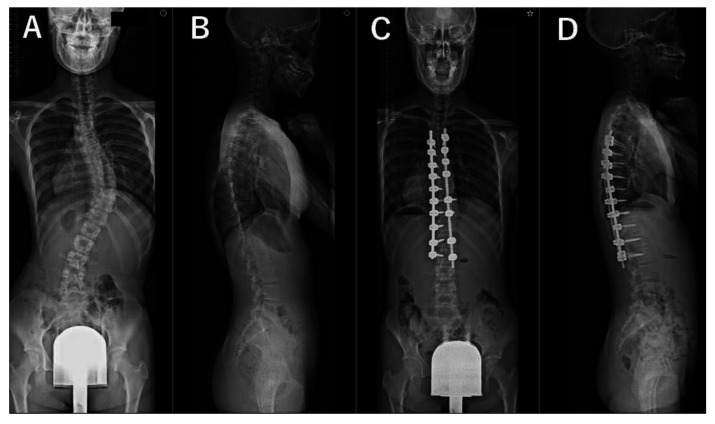
Pre- (**A**,**B**) and post- (**C**,**D**) operative radiographs of representative case with 12-year-old female patient. Cases of preoperative cervical lordosis changed to postoperative cervical lordosis. Cervical lordosis (1°, 23°), thoracic kyphosis (21°, 20°), lumbar lordosis (44°, 46°), T1 slope (13°, 19°), major thoracic curve (43°, 2°). (Preoperative, postoperative).

**Figure 4:**
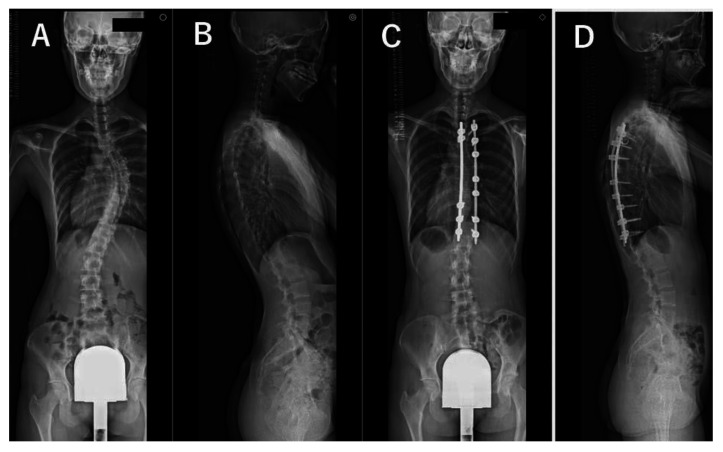
Pre- (**A**,**B**) and post- (**C**,**D**) operative radiographs of representative case with 20-year-old female patient. Cases of preoperative cervical hyperkyphosis changed to postoperative cervical lordosis. Cervical lordosis (−15°, 3°), thoracic kyphosis (20°, 28°), lumbar lordosis (59°, 52°), T1 slope (9°, 15°), major thoracic curve (51°, 17°). (Preoperative, postoperative).

For all patients, standing whole spine radiographs taken preoperatively and at 2 years postoperatively as a part of the routine examination were evaluated by a trained orthopedic surgeon who was not involved in the surgeries. The lateral spine radiographs were taken in a standing position with fists on the clavicles and by asking the patient to make a horizontal gaze (Figure 1, Figure 2, Figure 3 and Figure 4). We evaluated the main thoracic (MT) curves, T1 slope, cervical lordosis (C2-7 angle), TK (T5-12 angle), LL (L1-S1 angle), and each domain of the Scoliosis Research Society–22r Patient Questionnaire (SRS–22r) findings preoperatively and 2 years postoperatively. For the cervical and lumbar spine, lordotic alignment was presented with a positive value, and kyphotic alignment was presented with a negative value. On the other hand, for the thoracic spine, lordotic alignment was presented with a negative value, and kyphotic alignment was presented with a positive value. To investigate the impact of cervical kyphosis on clinical outcomes after AIS surgery, the patients were divided into two groups depending on the CSA, according to a previous report [12]: the cervical hyperkyphosis group: patients whose cervical lordosis at the 2-year follow-up was <−10°, non-cervical hyperkyphosis group: patients whose cervical lordosis was ≥−10°.

Informed consent was obtained from all patients and guardians. The protocol for this study has been approved by the institutional review board of our university hospital (approval number: 5851).

### Statistical Analysis

To measure the differences between the two groups, we used the t-test for continuous variables. The level of significance was set at *p* < 0.05. The relationship between Δcervical lordosis and ΔTK was assessed by Spearman’s rank correlation coefficient. To elucidate the associated factors for postoperative cervical hyperkyphosis at 2 years after surgery, we employed univariate and multivariate logistic analyses. In the multivariate analysis, the presence of cervical hyperkyphosis was the response variable, and age as well as those with *p* < 0.20 in the univariate analysis were the explanatory variables. All statistical analyses were conducted using SPSS (version 22.0, IBM, Chicago, IL, USA). All data except for Δ are expressed as the mean ± standard deviation. The data of Δ are expressed as the mean ± standard error.

## 3. Results

### 3.1. Demographics

Forty-five consecutive AIS patients (4 males and 41 females) who underwent PSF for Lenke type 1 and 2 curves were included. All patients completed the 2-year follow-up. The mean age at the time of surgery was 15.9 ± 2.7 years (range: 11–23 years). Lenke type was 1A in 29 patients, 1B in 5 patients, 1C in 6 patients, 2A in 3 patients, and 2B in 2 patients. The preoperative mean MT curve and mean TK were 52° ± 9°and 22° ± 11°, respectively. Before surgery, cervical kyphosis (cervical lordosis < 0°) was present in 40 patients (89% of the total), of whom cervical kyphosis was present in 27 patients (60% of the total). There were no significant differences in age, sex, or Lenke type between the hyperkyphosis and the non-hyperkyphosis groups.

### 3.2. SRS-22r Evaluations

In the comparison of each domain of SRS-22r before and after surgery, pain, self-image, mental health, and subtotal improved significantly (Table 1). There was no significant difference in function (Table 1). Comparing each domain of SRS–22r between the cervical hyperkyphosis and the non-hyperkyphosis groups, pain, mental health, and subtotal were significantly lower preoperatively in the cervical hyperkyphosis group (Table 2). At 2 years after surgery, mental health, subtotal, and total were significantly lower in the cervical hyperkyphosis group (Table 3). Pain tended to be worse in the cervical hyperkyphosis group (Table 3). Self-image and satisfaction at 2 years after surgery did not reach 4 points on average in the cervical hyperkyphosis group (Table 3).

### 3.3. Radiographic Evaluations

**Figure 5:**
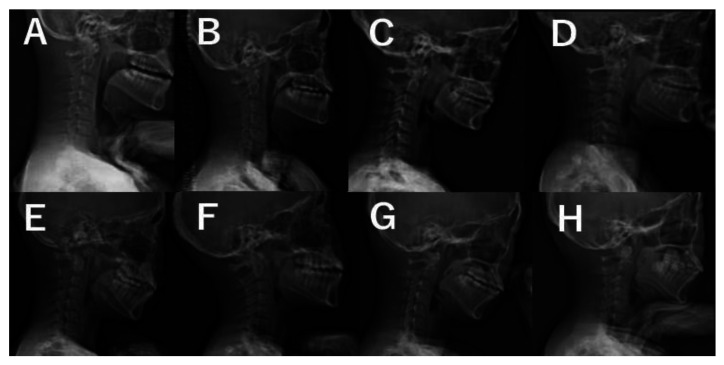
Pre- (**A**,**C**,**E**,**G**) and post- (**B**,**D**,**F**,**H**) operative magnified radiographs of the cervical spine from the cases in Figure 1 (**A**,**B**), Figure 2 (**C**,**D**), Figure 3 (**E**,**F**), and Figure 4 (**G**,**H**).

The MT curve was significantly corrected from 52° ± 9° preoperatively to 21° ± 5° at 2 years after surgery. The TK increased significantly from 22° ± 11° preoperatively to 32° ± 7° 2 years after surgery. Mean cervical lordosis significantly increased from −11° ± 10° preoperatively to −5° ± 11° postoperatively (*p* < 0.001); however, 33 of the 45 cases (73%) still had cervical kyphosis 2 years after surgery. Two years after surgery, there were 12 cases (27%) in the cervical hyperkyphosis group, all female. We present antero-posterior and lateral plain radiographs of the whole spine of representative cases in Figure 1, Figure 2, Figure 3 and Figure 4. Although preoperative CSA varies, postoperative CSA generally improves. Figure 5 shows magnified radiographs of the cervical spine from the cases in Figure 1, Figure 2, Figure 3 and Figure 4.

The pre-, postoperative, and changes in spinal alignments are summarized in Table 4. In addition, we investigated the correlation between ΔTK and Δcervical lordosis and found a significant correlation (r = 0.298, *p* = 0.046).

We performed a statistical analysis to clarify the factors associated with cervical hyperkyphosis 2 years after surgery. Univariate analysis comparing the cervical hyperkyphosis and the non-hyperkyphosis groups revealed significant differences in preoperative cervical lordosis, Δcervical lordosis, and ΔT1 slope (Table 5). In our multivariate logistic regression model, we therefore included age, preoperative cervical lordosis, ΔT1 slope, and ΔTK as covariates while treating the occurrence of postoperative cervical hyperkyphosis as dependent factors. Sex was excluded from this analysis as the cervical hyperkyphosis group 2 years after surgery consisted of females only. We found that preoperative larger cervical kyphosis (OR = 18, 95% CI: 1.7–195, *p* = 0.015), smaller ΔTK (OR = 6.0, 95% CI: 1.1–33, *p* = 0.043), and smaller ΔT1 slope (OR = 51, 95% CI: 2.8–961, *p* = 0.008) were independent associated factors for postoperative cervical hyperkyphosis (Table 5).

Our study included nine young adults aged 19 to 23 years old. In order to confirm whether these young adult cases had some impact on the results of the present study, we performed similar analyses with the exception of these nine cases of young adults and found similar results. These results are presented as supplement data in Tables S1–S5.

## 4. Discussion

In the present study, we identified that cervical kyphosis (cervical lordosis < 0°) was present in around 90% of patients and cervical hyperkyphosis (cervical lordosis < −10°) in 60% of patients before surgery in patients with AIS. AIS corrective surgery significantly improved cervical kyphosis after surgery, but it was confirmed that cervical kyphosis and cervical hyperkyphosis remained in about 70% of all cases and about 30% of all cases at 2 years after surgery, respectively. Comparing each domain of SRS–22r between the cervical hyperkyphosis and the non-hyperkyphosis groups, pain, mental health, and subtotal were significantly lower preoperatively in the cervical hyperkyphosis group (Table 2). At 2 years after surgery, mental health, subtotal, and total were significantly lower in the cervical hyperkyphosis group (Table 3). In addition, we found that preoperative larger cervical kyphosis (OR = 18, 95% CI: 1.7–195, *p* = 0.015), smaller ΔTK (OR = 6.0, 95% CI: 1.1–33, *p* = 0.043), and smaller ΔT1 slope (OR = 51, 95% CI: 2.8–961, *p* = 0.008) were independent associated factors for postoperative cervical hyperkyphosis (Table 5).

For a long time, scoliosis correction surgery in AIS has focused on the correction of coronal imbalance. Subsequently, surgeons are keenly aware that restoring TK is as important as correcting coronal imbalance. In recent years, in addition, surgical impacts on the sagittal curvature, especially CSA, have gained increasing attention [12,13,14,15,16,17,18,19]. However, we have not yet reached the setting of the ideal spinal sagittal alignment that corrective surgery for AIS should aim for.

Hilibrand et al. [20] first examined the sagittal alignment of the cervical spine in 38 patients with AIS. They identified a significant correlation between preoperative cervical lordosis and preoperative TK. Hilibrand et al. reported that the incidence of cervical kyphosis was 34 of 39 (89%) and concluded that patients with AIS developed lordosis within the thoracic spine, followed by compensatory cervical and lumbar kyphosis [20]. Consistent with this report, in the present study, we confirmed that the preoperative incidence of cervical kyphosis and cervical hyperkyphosis was approximately 90% and 60%, respectively. These results showed that, in AIS, more cases than we thought had cervical kyphosis before surgery. Subsequently, the relationship between cervical spine alignment and global spine alignment in the coronal and sagittal planes was reported by Mac-Thiong et al. [21]. These accumulated findings suggest that it is necessary to be conscious of the alignment of the global spine, not just local alignment.

It has been reported that the incidence of cervical kyphosis in AIS patients is higher than in healthy individuals [9,10,11]. A cervical lordosis in healthy individuals aged 10 to 20 years has been reported to range from 0° to 10° [22,23,24], while that of patients with AIS ranges from −10° to about 0° [20,25,26,27]. Few reports have directly compared cervical sagittal alignment in AIS patients and age-matched healthy controls. The report of Hiyama et al. [28] is one of the few reports of direct comparisons of cervical sagittal alignment in patients with 42 AIS and in age-matched 24 healthy controls. They found that the preoperative cervical lordotic angle was significantly more kyphotic in patients with AIS (−8.9° ± 16.1°) than in healthy individuals (2.5° ± 15.0°) (*p* < 0.01). However, this survey is limited to Lenke type 1 AIS only, and there is a limitation that other types of AIS have not been surveyed. Subsequently, Cho et al. investigated 318 cases of AIS, including Lenke types 1, 2, 3, and 6 [14]. They reported that 67.0% of AIS patients showed preoperative cervical kyphotic alignment. They found that postoperative CSA tended to improve, but cervical kyphosis remained after surgical treatment in more than 50% of cases [14]. Intriguingly, they found no significant difference in the postoperative changes in cervical lordotic angle by curve type. Consistent with these reports, in the present study, we also found that the cervical spine in AIS patients was in kyphotic alignment (preoperative mean cervical lordosis: −11° ± 10°). Although CSA showed significant improvement by PSF, postoperative cervical kyphosis and cervical hyperkyphosis remained at approximately 70% and approximately 25%, respectively. In the present study, we investigated not only Lenke type 1 but also Lenke type 2 AIS. Future studies with more cases are necessary, such as to determine whether there is a difference depending on the Lenke types.

Recent investigations revealed a correlation between the loss of normal TK and the development of cervical kyphosis [29,30]. Winter et al. [30] reported that cervical kyphosis in AIS is a compensatory phenomenon accompanied by loss of TK. However, the evidence regarding reciprocal changes in cervical alignment after corrective surgery for thoracic scoliosis is inadequate [12]. In the present study, we confirmed the improvement of CSA by reciprocal change after PSF for Lenke types 1 and 2 of AIS. In addition, there was a significant correlation between ΔTK and Δcervical lordosis. A similar improvement in CSA was reported in cases with a significant increase in TK after the surgical treatment [12,28,31,32], but not in cases without a significant increase in TK [9,16,33] after the surgical treatment. One of the likely reasons for the differences in TK transitions in previous reports is the choice of surgical procedure. Management of AIS is still controversial, and various surgical techniques are commonly used depending on the surgeon. While the all-pedicle screw construct provides strong fixation and excellent correction of coronal balance compared to other procedures such as the hybrid construct [34], it has been reported to cause postoperative flatback due to a loss of thoracic kyphosis [4]. On the other hand, there is an opinion that the hybrid construct is advantageous for obtaining TK but less likely to prevent the crankshaft phenomenon that occurs during remaining spinal growth when compared to the all-pedicle screws construct [35]. We must continue to search for ideal surgical procedures that make it possible to simultaneously improve coronal balance and restore adequate TK. Clement et al. also confirmed a strong correlation between TK gain and cervical lordosis gain (coefficient = 0.86) [36]. These findings support the opinion that cervical kyphosis in AIS is a compensatory phenomenon associated with flattening of the thoracic spine. It also suggests that restoration of TK in AIS surgery is also important for improving CSA. Zhang et al. advocated that the sagittal compensatory mechanism of the cervical spine before surgery was different from that after surgery [17]. That is, CSA was only determined by the regional sagittal profile of the thoracic spine (T1 slope and upper TK) before surgery, whereas it was strongly or moderately correlated with both the regional and global sagittal thoracic profiles, including T1 slope, upper TK, and global TK, after surgery. Further research is needed on the sagittal compensatory mechanism of the cervical spine in AIS. This should provide a clue to the ideal alignment setting that we should aim for during surgical treatment.

CSA has been reported to be associated with function and QOL. Patients with neck pain have cervical hypolordosis compared to healthy subjects [37,38]. Cervical kyphosis is also likely to play a substantial role in the development of cervical myelopathy [39,40]. However, the relationship between CSA and clinical outcomes, including QOL, in AIS is limited and controversial [41]. In AIS patients, frequent long-term neck pain after surgical treatment is correlated with CSA [42,43] and a significant relationship is found between postoperative cervical sagittal parameters and health-related QOL scores [41,43,44,45]. On the other hand, Cho et al. reported that CSA improved after corrective surgery for AIS, but there was no significant association between clinical outcomes and CSA [14]. Hayashi et al. [12] also found that the cervical lordotic angle increased significantly from preoperatively to 2 years after surgery; however, they found no difference in the clinical outcomes regardless of cervical hyperkyphosis. In this study, to clarify the effect of cervical kyphosis on QOL in AIS surgery, we focused on cervical hyperkyphosis, which exhibits cervical lordosis <−10° among cervical kyphosis, and found that the cervical hyperkyphosis group had significantly lower pain, mental health, and subtotal scores preoperatively. At 2 years after surgery, the cervical hyperkyphosis group tended to have worse pain scores and had significantly lower scores in some domains of SRS–22r (mental health, subtotal, and total). Pain and mental health are affected by many factors, and it is difficult to conclude that they are purely the result of spinal alignment alone. One of the major advances in recent years in the field of spinal surgery is the treatment of adult spinal deformities. In the surgical treatment of adult spinal deformities, it has been pointed out that sagittal balance correction is markedly important for better QOL [46]. Given the importance of postoperative sagittal global alignment in terms of QOL scores in patients with adult spinal deformities [47], postoperative sagittal global alignment, including the cervical spine, should also be important for patients with AIS. Further studies are needed to clarify the true impact of CSA on QOL in AIS.

So, is residual cervical kyphosis after scoliosis surgery predictable? Accumulating results showed that cervical alignment is strongly influenced by other sagittal parameters; that is, parameters of the sagittal plane, such as cervical lordosis, TK, and LL, are all closely related to each other [12,27]. Therefore, examining the relationship between multiple variables simultaneously may shed light on this issue. Hayashi et al. [12] evaluated the independent risk factors of postoperative cervical hyperkyphosis using multivariate logistic regression analysis and found that preoperative cervical kyphosis (preoperative cervical lordotic angle < −5°, OR = 8.59) and preoperative TK < 10° (OR = 12.5) were independently associated factors without any potential interaction. Zhang et al. [17] performed a retrospective study on 44 thoracic AIS patients treated with PSF. This study employed at least 5 years of follow-up after surgery, which is longer than the 2-year follow-up used in many other publications. They identified the preoperative cervical lordosis and postoperative T1 slope as predictors of the ultimate CSA using stepwise multilinear regression analysis [17]. In addition, to provide a more practical way of predicting the ultimate CSA, they utilized the receiver operation characteristic (ROC) curve to identify the cut points for preoperative cervical lordosis and postoperative T1 slope. According to this analysis, they disclosed that preoperative cervical lordosis ≥ −4.5° was strongly predictive and postoperative T1 slope ≥ 11.3° was moderately predictive of the ultimate lordotic cervical alignment [17]. Zhu et al. [18] also identified that AIS patients with a thoracic inlet angle less than 62° would have a postoperative uncorrected or new onset of cervical kyphosis using the ROC curve. Clement et al. reported that 60% of the gain in TK is transferred to the gain in distal cervical lordosis [36]. Zhang et al. [17] reported that CSA was strongly or moderately correlated with both regional and global sagittal thoracic profiles, including T1 slope, upper TK, and global TK after surgery. In the present study, preoperative larger cervical kyphosis (OR = 18), smaller ΔT1 slope (OR = 6.0), and smaller ΔTK (OR = 51) were the independent significant associated factors for postoperative cervical hyperkyphosis. These findings suggest that restoration of TK, mainly in the proximal segment, leading to a gain of the T1 slope, has important implications for improving cervical lordosis. In today’s surgical treatment of AIS, the ideal TK that we should aim for has not been set. The findings we mentioned above may help us set the ideal TK we should aim for.

Like every study, this one has several limitations. First, the sample size was small, but the characteristics of postoperative cervical hyperkyphosis cases and independent associated factors for postoperative cervical hyperkyphosis could be disclosed. Second, the follow-up period of this study was short. Thus, it was not possible to determine whether residual postoperative cervical kyphosis in patients with AIS increases the risk of future development of neck pain and/or cervical myelopathy. More long-term studies are warranted to disclose these issues. Third, the CSA evaluated by whole-spine radiography tends to be affected by factors such as patient rotation, posture, and the angle at which the radiograph was obtained, which may have created some bias. Fourth, this study retrospectively analyzed prospectively collected patients’ data, which lacked data on corresponding healthy adolescents. Radiographic imaging of healthy subjects always presents the problem of medical exposure. Moreover, considering the effects on puberty, it is not a test that can be performed easily. In addition, QOL assessments other than SRS–22r, such as EuroQol 5 Dimensions 5 level, have not been assessed. Future detailed studies are needed to clarify whether cervical kyphosis after AIS surgery has a different impact on QOL compared to cervical kyphosis in age-matched healthy subjects. Since this survey was limited to Lenke types 1 and 2 of AIS, it is unclear whether similar results can be obtained for other types. To clarify this issue, a further survey of a large number of cases covering all Lenke types is necessary. Determination of an ideal TK for AIS patients is difficult; therefore, the ideal TK that should be aimed at during surgery cannot be set.

## 5. Conclusions

Cervical kyphosis in AIS patients is considered a compensatory change due to a hypokyphotic thoracic spine. In AIS, cervical kyphosis is observed before surgery, and although it is significantly improved by PSF, there are quite a few cases in which kyphosis remains after surgery. There is a possibility that residual cervical hyperkyphosis after AIS surgery may affect postoperative results, including QOL, and we believe that it is necessary to focus on global spinal alignment, including the cervical spine, in future treatments. From this point of view, we believe that it is necessary to aim for TK restoration that leads to the gain of the T1 slope during corrective surgery for AIS.

## Figures and Tables

**Table 1:** Comparison of SRS-22r before and after surgery in all participants.

SRS-22r Domain	Preoperative	Postoperative	*p*-Value
Function	4.59 ± 0.44	4.73 ± 0.26	0.05
Pain	4.30 ± 0.61	4.53 ± 0.45	0.01
Self-image	2.81 ± 0.50	4.00 ± 0.64	<0.001
Mental health	4.10 ± 0.64	4.31 ± 0.60	0.02
Subtotal	4.04 ± 0.41	4.40 ± 0.32	<0.001
Satisfaction	/	3.98 ± 0.72	/
Total	/	4.35 ± 0.33	/

Data are expressed as the mean ± standard deviation.

**Table 2:** Comparison of preoperative SRS-22r between hyperkyphosis and non-hyperkyphosis groups.

SRS-22r Domain	Hyperkyphosis	Non-Hyperkyphosis	*p*-Value
Function	4.53 ± 0.43	4.61 ± 0.44	0.63
Pain	4.00 ± 0.73	4.41 ± 0.53	0.047
Self-image	2.62 ± 0.56	2.88 ± 0.46	0.119
Mental health	3.77 ± 0.73	4.22 ± 0.57	0.036
Subtotal	3.80± 0.42	4.12 ± 0.37	0.018
Satisfaction	/	/	/
Total	/	/	/

Hyperkyphosis group: patients whose cervical lordosis at the 2-year follow-up < −10°, non-hyperkyphosis group: patients whose cervical lordosis at the 2-year follow-up ≥ −10°. Data are expressed as the mean ± standard deviation.

**Table 3:** Comparison of SRS-22r at 2 years after surgery between hyperkyphosis and non-hyperkyphosis groups.

SRS-22r Domain	Hyperkyphosis	Non-Hyperkyphosis	*p*-Value
Function	4.75 ± 0.19	4.73 ± 0.28	0.80
Pain	4.32 ± 0.43	4.61 ± 0.44	0.05
Self-image	3.82 ± 0.62	4.07 ± 0.65	0.25
Mental health	4.00 ± 0.77	4.42 ± 0.49	0.03
Subtotal	4.22 ± 0.36	4.46 ± 0.28	0.02
Satisfaction	3.75 ± 0.75	4.06 ± 0.71	0.21
Total	4.18 ± 0.35	4.42 ± 0.30	0.02

Hyperkyphosis group: patients whose cervical lordosis at the 2-year follow-up < −10°, non-hyperkyphosis group: patients whose cervical lordosis at the 2-year follow-up ≥ −10°. Data are expressed as the mean ± standard deviation.

**Table 4:** The pre-, postoperative, and changes in spinal alignments.

Variables	Preoperative	Postoperative	Δ	*p*-Value
CL (°)	−11 ± 10	−5 ± 11	7 ± 2	<0.001
TK (°)	22 ± 11	32 ± 7	10 ± 1	<0.001
LL (°)	54 ± 11	56 ± 11	2 ± 1	0.13
MT curve (°)	52 ± 9	21 ± 5	−32 ± 1	<0.001
T1 slope (°)	16 ± 6	20 ± 7	4 ± 1	<0.001

CL: cervical lordosis, TK: thoracic kyphosis, LL: lumbar lordosis, MT: major thoracic. Data except for Δ are expressed as the mean ± standard deviation. Data of Δ are expressed as mean ± standard error.

**Table 5:** Univariate and multivariate analyzes of association between hyperkyphosis at 2 years postoperative and age and other candidate factors.

Candidate	Univariate Analysis		Multivariate Analysis	
	OR (95% CI)	*p*-Value	OR (95% CI)	*p*-Value
Pre CL (−10°)	3.5 (1.3–9.7)	0.015	18 (1.7- 195)	0.015
Pre TK (−10°)	1.2 (0.67–2.2)	0.50		
Pre LL (−10°)	0.69 (0.37–1.3)	0.24		
Pre T1 slope (−10°)	1.5 (0.48–4.5)	0.50		
ΔCL (−10°)	3.0 (1.1–8.1)	0.026		
ΔTK (−10°)	1.7 (0.82–3.7)	0.15	6.0 (1.1–33)	0.043
ΔLL (−10°)	1.0 (0.49–2.2)	0.91		
ΔT1 slope (−10°)	7.7 (1.6–38)	0.012	51 (2.8–961)	0.008
Age (+1 year)	1.1 (0.87–1.4)	0.43	1.3 (0.84–2.1)	0.22

OR: odds ratio, CI: confidence interval, Pre: preoperative, CL: cervical lordosis, TK: thoracic kyphosis, LL: lumbar lordosis.

## Data Availability

The datasets generated during and/or analyzed during the current study are available from the corresponding author on reasonable request.

## Supplementary Materials

The following supporting information can be downloaded at: https://www.mdpi.com/article/10.3390/jcm12175599/s1, Table S1. Comparison of SRS-22r before and after surgery in participants in aged 18 years or younger. Table S2. Comparison of preoperative SRS-22r between hyperkyphosis and non-hyperkyphosis groups in aged 18 years or younger. hyperkyphosis group: patients whose cervical lordosis at the 2-year follow-up ≦ −10°, non-hyperkyphosis group: patients whose cervical lordosis at the 2-year follow-up > −10°. Data are expressed as the mean ± standard deviation. Table S3. Comparison of SRS-22r at 2 years after surgery between hyperkyphosis and non-hyperkyphosis groups in aged 18 years or younger. hyperkyphosis group: patients whose cervical lordosis at the 2-year follow-up ≦ −10°, non-hyperkyphosis group: patients whose cervical lordosis at the 2-year follow-up > −10°. Data are expressed as the mean ± standard deviation. Table S4. The pre-, postoperative, and changes in spinal alignments in aged 18 years or younger. CL: cervical lordosis, TK: thoracic kyphosis, LL: lumbar lordosis, MT: major thoracic. Data except for Δ are expressed as the mean ± standard deviation. Data of Δ is expressed as mean ± standard error. Table S5. Univariate and multivariate analyzes of association between hyperkyphosis at 2 years postoperative and age and other candidate factors in aged 18 years or younger. OR: Odds ratio, CI: confidence interval, Pre: preoperative, The lordosis alignment is shown as a positive value for the cervical and lumbar spine and a negative value for the thoracic spine.
