# Supplementary material for: Reciprocal Change of Cervical Spine after Posterior Spinal Fusion for Lenke Type 1 and 2 Adolescent Idiopathic Scoliosis
Source: J Clin Med. 2023 Aug 28;12(17):5599. doi: 10.3390/jcm12175599 (PMC10488443; doi:10.3390/jcm12175599)
Supplement: Supplementary file 1 [file jcm-12-05599-s001.zip › jcm-2561696-supplementary.pdf]

SRS-22r domain	Preoperative	Postoperative	*p*-value
Function	4.58 ± 0.45	4.73 ± 0.24	0.098
Pain	4.27 ± 0.66	4.54 ± 0.47	0.021
Self-image	2.79 ± 0.53	3.96 ± 0.66	<0.001
Mental health	4.13 ± 0.67	4.35 ± 0.62	0.046
Subtotal	4.05 ± 0.44	4.40 ± 0.33	< 0.001
Satisfaction	/	3.97 ± 0.76	/
Total	/	4.35 ± 0.34	/

Table S1. Comparison of SRS-22r before and after surgery in participants in aged 18 years or younger.

SRS-22r domain	hyperkyphosis	non-hyperkyphosis	*p*-value
Function	4.47 ± 0.47	4.62 ± 0.45	0.38
Pain	3.84 ± 0.75	4.41 ± 0.58	0.025
Self-image	2.51 ± 0.59	2.88 ± 0.49	0.070
Mental health	3.62 ± 0.79	4.30 ± 0.53	0. 006
Subtotal	3.69 ± 0.42	4.17 ± 0.38	0.003
Satisfaction	/	/	/
Total	/	/	/

Table S2. Comparison of preoperative SRS-22r between hyperkyphosis and non-hyperkyphosis groups in aged 18 years or younger.

hyperkyphosis group: patients whose cervical lordosis at the 2-year follow-up ≦ -10°, non-hyperkyphosis group: patients whose cervical lordosis at the 2-year follow-up > -10°. Data are expressed as the mean ± standard deviation.

SRS-22r domain	hyperkyphosis	non-hyperkyphosis	*p*-value
Function	4.73 ± 0.20	4.73 ± 0.26	0.94
Pain	4.22 ± 0.42	4.64 ± 0.45	0.018
Self-image	3.69 ± 0.58	4.05 ± 0.67	0.16
Mental health	3.84 ± 0.80	4.52 ± 0.45	0.003
Subtotal	4.12 ± 0.36	4.49 ± 0.27	0.003
Satisfaction	3.72 ± 0.87	4.06 ± 0.71	0.26
Total	4.09 ± 0.36	4.44 ± 0.30	0.006

Table S3. Comparison of SRS-22r at 2 years after surgery between hyperkyphosis and non-hyperkyphosis groups in aged 18 years or younger.

hyperkyphosis group: patients whose cervical lordosis at the 2-year follow-up ≦ -10°, non-hyperkyphosis group: patients whose cervical lordosis at the 2-year follow-up > -10°. Data are expressed as the mean ± standard deviation.

Variables	Preoperative	Postoperative	Δ	*p*-value
CL (°)	-11 ± 10	-4 ± 10	7 ± 2	< 0.001
TK (°)	22 ± 12	31 ± 7	9 ± 2	< 0.001
LL (°)	54 ± 11	54 ± 11	1 ± 1	0.53
MT Cobb (°)	54 ± 10	21 ± 6	-33 ± 1	< 0.001
T1-slope (°)	17 ± 6	20 ± 7	4 ± 1	< 0.001

Table S4. The pre-, postoperative, and changes in spinal alignments in aged 18 years or younger.

CL: cervical lordosis, TK: thoracic kyphosis, LL: lumbar lordosis, MT: major thoracic. Data except for Δ are expressed as the mean ± standard deviation. Data of Δ is expressed as mean ± standard error.

Candidate	Univariate analysis		Multivariate analysis	
	OR (95%CI)	*p*-value	OR (95%CI)	*p*-value
Pre CL (-10°)	3.5 (1.1-11)	0.032	25 (1.1- 545)	0.041
Pre TK (-10°)	1.4 (0.73-2.8)	0.29		
Pre LL (-10°)	0.81 (0.40-1.6)	0.55		
Pre T1 slope (-10°)	0.98 (0.26-3.7)	0.97		
ΔCL (-10°)	2.1 (0.87-5.1)	0.10		
ΔTK (-10°)	1.5 (0.66-3.3)	0.34	6.8 (0.8-56)	0.079
ΔLL (-10°)	0.81 (0.40-1.6)	0.55		
ΔT1-slope (-10°)	7.8 (1.3-47)	0.025	112 (2.5-5059)	0.015
Age (+1 year)	1.3 (0.80- 2.1)	0.28	1.5 (0.55-4.1)	0.42

Table S5. Univariate and multivariate analyzes of association between hyperkyphosis at 2 years postoperative and age and other candidate factors in aged 18 years or younger.

OR: Odds ratio, CI: confidence interval, Pre: preoperative,

The lordosis alignment is shown as a positive value for the cervical and lumbar spine and a negative value for the thoracic spine.
